# Post-Script

**Published:** 1903-12

**Authors:** 


### POST-SCRIPT.

It is due to Dr. McDaniel to say that after his paper was ready for press, and it was too late to withdraw or alter it, he wrote, requesting me to suppress it, stating that it was incomplete. I informed him that it was too late, and remarked that the cases resulting from Mrs. Kurka's case (two at Hondo and three at San Antonio) convinced me that the disease was yellow fever (of which I had been skeptical because it did not spread). And I suggested that it would be in order to explain by whose permission and authority a yellow fever corpse was shipped, and a nurse from the room of a fatal case of yellow fever was permitted to leave San Antonio? I publish below his reply.

I can not understand the line of reasoning by which it is sought to make those who questioned the diagnosis of yellow fever *responsible for the quarantine.* It was clearly the State Health Officer's duty to put on quarantine as soon as he was convinced of the presence of the infection in San Antonio (and the Kurka cases and sequels left no room to doubt it), and he did this duty promptly, and yet the majority of the profession there—those who say they were co-operating with him—seem to think that but for the *antis* there would have been no quarantine.

To my mind these cases demonstrated the fact that the infection was *not* spread by the mosquito, and also make clear the diagnosis; but why the fever did not become epidemic all over Texas will ever remain a mystery, for the gap was not put up until the cow had gotten out; that is to say, the infection evidently existed in San Antonio long before Mrs. Kurka was taken sick (October 6th) and before the assembling in San Antonio of thousands of visitors to the Fair (October 18th to 28th), and their subsequent stampede, all of which occurred before quarantine was put on. It is sheer nonsense to say that yellow fever can not become epidemic in San Antonio; but *why* it did not is a mystery. It was there, and there are many s. f. mosquitoes there, and as the disease was not at first recognized, of course no means were resorted to to defeat the mosquito or to prevent the spread of the infection by persons and things by atmospheric infection.

### DR. M'DANIEL'S LETTER.

SAN ANTONIO, December 3, 1903.

*Dr. F. E. Daniel, Austin, Texas.*

DEAR SIR: Yours to hand this morning, and I am really sorry that I did not get to suppress my article from your paper, first, because it was incomplete, and, as you say in your letter, an explanation is in order why Mrs. Kurka's body was shipped out of San Antonio and the nurse allowed to go with her. This explanation should have been in that article. And, second, I failed to state that Dr. Tabor was called to Hondo* instead of San Antonio, and merely said that this was the case that Dr. Tabor was called to, which statement, also, might demand an explanation. I do not care to have it inferred that I had called Dr. Tabor, and if it is not too late, would be glad to cut that part out of the article. I refused to sign the death certificate for Mrs. Kurka and insisted, at the time, that the case was suspicious, and that it was at least prudent to quarantine the premises for five days. The city health physician, Dr. Burg, as well as the board of health, disagreed with me, and Dr. Burg, gave orders, over my head, to have the body shipped out. This was the unfortunate occurrence that brought about the quarantine, and, while I have been condemned for reporting the first case, I really did everything in the world to prevent this body being shipped out, and had that not been done, the quarantine never would have occurred.

Very respectfully,
A. S. McDaniel, M. D.

---

Letter to the "Red-Back."—Dr. A. M. Denman of Lufkin renews his subscription, and pays five years in advance. He writes:

*F. E. Daniel, Editor "Red-Back," Austin, Texas.*

Dear Sir: I agree with you; a doctor who can't or won't pay for his medical journal, even two or three years in advance, ought to move or go into some other kind of business. A doctor who won't pay his bills ought not kick if his patrons do not pay him. Pay your bills, ye Texas doctors, and everybody will respect you and appreciate you work by paying you. I have paid for the "Red-Back" in advance; this is the second time.

Of the two bad paymasters, I had rather be the one who pays in advance. If Betty and the baby get cold and hungry, write me and I will chip in to buy them some socks and some grub.

Yours truly,
A. M. Denman.

---

*The doctor stated it correctly—see his letter.—Daniel.

